# Understanding differences in mental health service use by men: an intersectional analysis of routine data

**DOI:** 10.1007/s00127-022-02256-4

**Published:** 2022-03-22

**Authors:** Natasha Smyth, Joshua E. J. Buckman, Syed A. Naqvi, Elisa Aguirre, Ana Cardoso, Stephen Pilling, Rob Saunders

**Affiliations:** 1 Centre for Outcomes Research and Effectiveness, Research Department of Clinical, Educational and Health Psychology, University College London, London, WC1E 7HB UK; 2 iCope, Camden and Islington Psychological Therapies Services, Camden & Islington NHS Foundation Trust, St Pancras Hospital, London, UK; 3 North East London NHS Foundation Trust, London, UK; 4 Camden and Islington NHS Foundation Trust, London, UK

**Keywords:** Men’s mental health, Intersectionality, Social determinants, Engagement, Community mental health, Utilisation

## Abstract

**Purpose:**

Rates of help-seeking for common mental health problems are lower for men, but less is known about patterns of engagement once they are in contact with services. Previous research has been limited in its ability to understand the intersection between service user characteristics and engagement. This study compared analytic approaches to investigate intersectional associations between sociodemographic and socioeconomic indicators and use of psychological treatment services by men.

**Method:**

Data from 9,904 male service users attending two psychological treatment services in London were analysed. The association between ethnicity, sexual orientation, religious affiliation and employment status of service users and service use outcomes was explored using multinomial logistic regression and latent class analysis (LCA).

**Results:**

Being from a minoritised ethnic background, of Muslim faith, being unemployed, and living in the most deprived neighbourhoods were associated with greater risk of not commencing or completing treatment. Seven classes were identified in LCA, with men predominately differentiated by self-reported ethnicity and religion. Compared with the ‘White British, non-religious’ class, the ‘Asian Muslim’ class and the ‘minoritised ethnic, non-religious’ class were at higher risk of disengagement, whilst the ‘Asian, other religion’ class were at higher risk of being referred elsewhere rather than completing initiated treatment.

**Conclusions:**

There were significant inequalities in engagement by men associated with ethnicity, religion and socioeconomic status. Compared with the regression models, further nuance was apparent in LCA regarding the intersection of gender, religion and ethnicity. Identifying groups at greater risk of discontinuation of treatment could inform more personalised pathways through care.

**Supplementary Information:**

The online version contains supplementary material available at 10.1007/s00127-022-02256-4.

## Introduction

The prevalence of specific mental health problems differs between men and women. For example, men are both more likely to be diagnosed with substance use disorders (SUD) and to complete suicide than women [1–3], and both the prevalence of common mental health disorders (CMD) (such as anxiety disorders and depression) and rate of treatment-seeking for mental health problems is considerably higher in women [3–6]. Some of these gender differences might be explained by a perceived incompatibility of expressing and seeking help for mental distress whilst conforming with masculine traits such as stoicism and self-reliance, meaning mental distress could be judged (by self or others) as a sign of inherent weakness [4, 7, 8]. However, studies investigating the link between conformity with masculine norms and help-seeking have typically been conducted with healthy, White, male students or general community members with self-reported diagnoses [7]. As such, within group differences of men accessing clinical services are not well understood, and the discourse on the role of masculinity has often been “victim-blaming” (i.e. the man’s stubbornness fuels their lack of service use) [9]. This may deflect attention away from the potential ways in which being a man intersects with other facets of social identity in both health promoting and health depleting ways [9]. Even less is known about men’s mental health service use once contact has been initiated, with the quantitative literature predominately focussing on measuring help-seeking, typically operationalised by whether professional help has been sought in the past year or during the person’s lifetime [10]. Few studies which do investigate ‘disengagement’, discriminate between those service users who make an independent decision to discontinue treatment compared to those that discontinue in agreement with their clinician, despite this being an important distinction [11]. As such, there is little knowledge of the pathway through care men experience once they have sought help. Qualitative studies suggest a tendency for men to: (i) seek help only after exhausting all other perceived avenues for support; and (ii) struggle to fully engage in the therapeutic process [12]. This indicates that gender shapes men’s experience of using services throughout the treatment pathway.

The literature on social determinants of mental health service use indicates that, in addition to gender, other service user characteristics such as being from a minoritised ethnic group, both being younger and older in age are associated with reduced levels of service use [13–17], whereas being Lesbian, Gay, Bisexual (LGB), attending religious services [18–21], and lower socioeconomic status (SES) [14, 22, 23] are associated with increased use. However, this literature treats social determinants as single factors which exert independent effects. Yet, the experience of being a gay Christian Black man, for example, may not be adequately captured by exploring the effects of sexual orientation, religious affiliation, ethnicity, and gender, independently and instead, it may be important to consider the intersectionality of these factors. Intersectionality draws attention to the multiple and mutually constitutive social positions occupied by people, seeking to uncover the corresponding levels of power or disadvantage conferred to the individual [24]. The theory posits that exploration of particular combinations of social positions exert influence that is distinct from the sum of those positions [25].

Few studies have sought to investigate men’s mental health service use from an intersectional perspective. However, those that have, reported intersectional influences which have led to a more nuanced understanding. For example Parent et al. 2018 analysed mental health help-seeking across three ethnic groups and found greater wealth (measured using an ‘income-poverty ratio’) was positively associated with help-seeking among White men, negatively associated with help-seeking among Black men and unrelated to help-seeking among Mexican American men [26]. This approach focussed on gender and ethnicity as groups of interest, however considering multiple sociodemographic and socioeconomic factors has the potential to further explore intersectionality in relation to men’s mental health service use.

To date, the intersectional perspective has more commonly been applied using qualitative methods. This may reflect (contested) perceptions of ill fit with quantitative epistemology and methodology [27, 28]. There is a need for more quantitative research to address intersectional questions to increase the scope of intersectional knowledge and advance thinking around which methods are most complementary. One potential quantitative method for exploring intersectionality is Latent Class Analysis (LCA) [29, 30], a data-driven clustering approach used to identify statistically distinct subgroups [31]. LCA aligns well with the intersectional approach because it allows for the simultaneous consideration of multiple interacting risk-factors which can be difficult to achieve using traditional multiple regression modelling. LCA has been used to identify groups at greater risk of developing CMD based upon individual social identity indicators (ethnicity, migrant status and multiple indicators of SES) [29] or identifying groups who are more or less likely to benefit from psychological therapy based upon demographic and self-reported symptom data [32], but at present not to identify groups with differential service use.

Identifying subgroups at risk of discontinuing from treatment might help reduce levels of unmet need among men and improve their outcomes. Both attending fewer treatment sessions and poor engagement with psychological interventions are associated with worse clinical outcomes and greater likelihood of needing additional care [33, 34], and there is mounting evidence that tailoring services to meet the needs of underserved groups can improve uptake and outcomes [35–37]. Discontinuation from treatment can occur in several ways, including the service user cancelling or failing to attend sessions, or the service not being deemed to be the most suitable to meet the service user’s needs. This might be a joint decision between the service user and treating clinician, or a clinician-led decision, or even one led by service managers. Discontinuation might include the service user being referred on to other services, but as this is likely to lead to continued service use it might be considered different from the two reasons above. Likewise, if a service user never attends sessions, or declines treatment with a service, it cannot be considered a form of discontinuation of treatment as no treatment was initiated. Addressing disparities among service users that discontinue care by disengaging, compared to the service being deemed not suitable for their needs may require different responses, so differentiating between the two may be of clinical value. Likewise, establishing whether discontinuation occurs prior, or subsequent to treatment commencing, may help inform service interventions to tackle the problem.

Given the novelty of exploring differential service use by men using LCA, this study compares traditional methods of regression modelling to LCA to explore associations. The aims of this study are to (1) explore the association between sociodemographic and socioeconomic indicators and use of psychological treatment services; (2) identify distinct subgroups of service users defined by sociodemographic and socioeconomic indicators and (3) to explore the associations between identified classes and service use.

## Methods

### Services and service users

Data for this study come from two community-based psychological treatment services in North East London. These Improving Access to Psychological Therapies (IAPT) services offer evidence-based psychological treatments for anxiety and depression. A key feature of IAPT is the central role of data collection for monitoring and evaluation purposes which has resulted in exceptionally high levels of data completeness [38]. As a national programme, activity levels and outcomes for all services are reported annually and can be compared. In 2017–2018, nationally 978,477 people attended an initial assessment appointment at an IAPT service in England [39]. Following which, the service was considered ‘unsuitable’ for service users in 28,733 (3%) cases so they did not proceed on to treatment, and 395,035 (40%) service users were recorded as ending after having had only one appointment. In that same year, 517,942 (53%) service users that were referred and assessed by IAPT services completed a course of treatment. Although not stated, the remaining 36,767 (4%) service users not accounted for in this breakdown may represent those with missing service use data. The reason for the end of an episode of care is defined within the IAPT programme by the treating clinician at the point of discharging a service user; if the service user has completed the scheduled or planned number of sessions they are determined to have completed treatment.

Service users self-identifying as male that had undergone an assessment and had been discharged from their episode of care (including those not entering treatment) with the two included IAPT services between October 2011 (when services were operational and data collection began) and February 2020, were included in the current study. Where service users had received multiple episodes of care, for example additional treatment episodes, only the first episode of care was included. Service users were excluded if there was no data on their reason for ending contact with the service following an initial assessment, as this is the outcome of interest for this study. The reason for missing outcome data was unclear, and comparison between those with and without outcome data indicated some differences between groups regarding ethnicity, sexuality, religion, and neighbourhood deprivation level (See Appendix A). A total of 9904 service users met inclusion criteria and were included in the analyses (see Appendix B for participant flow diagram).

### Measures

#### Indicator variables

The indicator variables used in this study are derived from routinely available data collected at an initial assessment with the services for all service users. Some variables were recoded and combined due to limited responses in some categories. These included self-reported:

- Ethnicity: categorised into UK Census categories: “White”, “Black”, “Mixed”, “Asian”, and “Other”.
- Sexual orientation: using categories “heterosexual” and “lesbian, gay, bisexual (LGB)”.
- Religious affiliation: using “no stated religion”, “Christian”, “Muslim”, and “Other”.
- Employment status: categorised into “unemployed” or “not-unemployed”. The unemployed group was comprised of those indicating that they are “unemployed and seeking work”, “unable to work due to sickness or disability” and those “not actively seeking work”. The not-unemployed group comprised of those indicating that they are either “employed”, “students”, “homemakers”, “volunteers not seeking work” or “retired”.
- Neighbourhood deprivation: using the English Indices of Multiple Deprivation (IMD) [40] which measure relative deprivation across multiple domains (including income, employment, education and housing) of small areas in England. IMD scores were calculated for individual service users based on their residential postcode (grouped by Lower Layer Super Output Area (LSOA). For the purposes of this study IMD scores were collapsed into quintiles.

### Outcomes

Two nominal categorical outcome variables were created using the reason recorded by the treating IAPT clinician for the end of each service user’s treatment episode. Following the initial assessment, service users were categorised into one of the following groups of “Assessment phase” outcomes: (i) ‘entered treatment’, (ii) ‘disengaged’, (iii) ‘service deemed unsuitable’. The ‘entered treatment’ group were those who went on to have at least 1 treatment session or were referred to another clinical service for treatment, as defined by the services [41]. Those who entered IAPT treatment were then categorised into the one of the following “Treatment phase” groups: (i) completed treatment, (ii) disengaged, (iii) referred elsewhere.

### Covariates

Depression and anxiety symptom scores at baseline, measured on the Patient Health Questionnaire 9-item [42] and the Generalised Anxiety Disorder scale 7-items [43] respectively, as well as service user age were included as continuous covariates in regression analysis, as these variables have been associated with outcomes, including engagement, in previous analyses using similar data [44, 45]. Data on each service user’s ‘problem descriptor’, used by the services as a proxy for diagnosis, were included as categorical covariate within regression analyses and grouped in accordance with conventions set by previous publications using IAPT datasets [46, 47].

### Statistical analysis

To answer the first aim, multinomial logistic regression models were constructed to explore associations between indicator variables and service use outcomes. The full sample (*n* = 9904) was used to test associations with the assessment phase outcomes. A smaller sample which included service users who entered treatment after the assessment phase (*n* = 6852) was used to test associations with the treatment phase outcomes. Given the relatively low levels of missing data, ‘missing’ was created as an additional value for categorical variables and included in the analyses so that service users with missing values were not subject to listwise deletion in the initial multinomial regression models [44]. For this analysis, the following models were estimated for both assessment and treatment phase outcomes, with each indicator variable: Model 1 tested the unadjusted association between each indicator variable and the outcomes; model 2 adjusted for age, problem descriptor and symptom severity; model 3 fully adjusted for all indicator variables and covariates.

To meet the second objective, LCA was conducted using ethnicity, sexual orientation, religious affiliation, and employment status as class indicators. Missing data on these indicator variables was not included as its own category and instead was managed using Full Information Maximum-Likelihood through the Expectation Maximisation (EM) algorithm [48]. Given that neighbourhood deprivation level represents an area, rather than individual level, variable it was not modelled in the LCA but was instead treated as a covariate alongside age in later regression models testing the association between the classes and service use outcomes. Selection on the optimum class solution was made in accordance with established criteria [32, 49–51] with full details provided in Appendix C. Once the final class solution was identified, multinomial logistic regression models were constructed to explore the associations between identified classes and service use outcomes. Model 1 included class (unadjusted) and model 2 was adjusted for age, problem descriptor, symptom severity and neighbourhood deprivation. Multinomial regression models were constructed in stata15 [52] with relative risk ratios (RRR) and 95% confidence intervals (95%CI) reported, and LCA was performed in Mplus V8 [53].

## Results

### Sample characteristics

Descriptive statistics of the sample are presented in Table 1. Nearly 41% of service users identified as belonging to a minoritised ethnicity group, 60% identified as religious, and 93% as heterosexual. Local area deprivation scores were high, compared to the national average. Following assessment, most service users started treatment (69%) with the remaining group either disengaging or the service was deemed unsuitable for them, in roughly equal numbers. Of the number of service users who entered treatment (*n* = 6852), 57% were considered to have completed their treatment episode, and 35% were considered to have disengaged from treatment. This figure is slightly higher than the national average of 53% for treatment completion amongst service users entering treatment in 2017–2018.

**Table 1:** Sample characteristics

	*n* (%)
Total sample	9904
Age (years)	
16–24	1513 (15.3%)
25–34	2665 (26.9%)
35–44	2206 (22.3%)
45–54	1903 (19.2%)
55–64	1109 (11.2%)
65 +	507 (5.1%)
Ethnicity	
White	5769 (58.3%)
Asian	2597 (26.2%)
Black	902 (9.1%)
Mixed	370 (3.7%)
Other	184 (1.9%)
Missing	81 (0.8%)
Sexual orientation	
Heterosexual	9234 (93.2%)
LGB	291 (2.9%)
Missing	378 (3.8%)
Religion	
No religion Christian	3609 (36.4%) 2910 (29.4%)
Muslim	1695 (17.1%)
Other	1294 (13.1%)
Missing	395 (4.0%)
Employment	
Not-unemployed	6502 (65.7%)
Unemployed	3288 (33.2%)
Missing	113 (1.1%)
IMD (quintiles)	
1 (Least deprived)	271 (2.7%)
2	870 (8.8%)
3	1915 (19.3%)
4	3301 (33.3%)
5 (Most deprived)	3277 (33.1%)
Missing	269 (2.7%)
Assessment phase	
Started treatment	6852 (69.2%)
Disengaged	1483 (15.0%)
Service deemed unsuitable	1569 (15.9%)
Treatment phase	
Assessment only	3052 (30.8%)
Treatment completed	3899 (39.4%)
Disengaged	2394 (24.2%)
Referred elsewhere	559 (5.6%)

	*n* (%)
Baseline severity score	
PHQ-9 mean (SD)	15.6 (6.4)
Missing	51 (0.5%)
GAD-7 mean (SD)	13.7 (5.2)
Missing	59 (0.6%)
Diagnosis	
Depression	6764 (68.3%)
Generalised Anxiety Disorder (GAD)	246 (2.5%)
Mixed Anxiety and Depression	492 (5.0%)
Obsessive–compulsive disorder (OCD)	93 (0.9%)
Post-traumatic stress disorder (PTSD)	294 (3.0%)
Phobic anxiety & Panic	520 (5.3%)
Severe mental illness (SMI)	8 (0.1%)
Unspecified anxiety disorder	578 (5.8%)
Missing	738 (7.5%)

### Service use patterns by social status indictors

The association between each indicator variable and assessment outcomes is presented in Table 2, and for treatment outcomes in Table 3.

**Table 2:** Associations between social status indicators and service use outcomes following the initial assessment

	Assessment phase outcome variables^1^					
	Disengaged			Service deemed unsuitable		
	Model 1	Model 2	Model 3	Model 1	Model 2	Model 3
	RRR (95% CI)	RRR (95% CI)	RRR (95% CI)	RRR (95% CI)	RRR (95% CI)	RRR (95% CI)
Ethnicity						
White	Ref.	Ref.	Ref.	Ref.	Ref.	Ref.
Asian	1.52 (1.33, 1.72) ***	1.39 (1.22, 1.58) ***	1.32 (1.07, 1.61) **	1.14 (1.01, 1.30) *	1.19 (1.04, 1.37)*	1.18 (0.95, 1.45)
Black	1.35 (1.11, 1.64) **	1.29 (1.06, 1.58) *	1.32 (1.07, 1.64) *	1.22 (1.01, 1.47) *	1.27 (1.04, 1.55)*	1.26 (1.01, 1.56)*
Mixed	1.15 (0.85, 1.56)	1.02 (0.75, 1.38)	0.99 (0.71, 1.37)	1.13 (0.85, 1.50)	1.13 (0.84, 1.53)	1.07 (0.77, 1.49)
Other	1.40 (0.94, 2.08)	1.21 (0.80, 1.82)	1.26 (0.81, 1.98)	1.04 (0.69, 1.58)	1.04 (0.67, 1.62)	1.01 (0.61, 1.65)
Sexuality						
Heterosexual	Ref.	Ref.	Ref.	Ref.	Ref.	Ref.
LGB	1.40 (1.03, 1.91) *	1.25 (0.92, 1.71)	1.30 (0.94, 1.80)	1.27 (0.93, 1.74)	1.22 (0.88, 1.70)	1.23 (0.87, 1.73)
Religion						
No religion	Ref.	Ref.	Ref.	Ref.	Ref.	Ref.
Christian	0.77 (0.67, 0.89) ***	0.90 (0.77, 1.04)	0.85 (0.72, 1.00) *	0.95 (0.83, 1.09)	0.98 (0.85, 1.13)	0.94 (0.80, 1.10)
Muslim	1.30 (1.12, 1.52) **	1.31 (1.12, 1.53) **	1.03 (0.83, 1.29)	1.02 (0.87, 1.20)	1.07 (0.90, 1.26)	0.93 (0.74, 1.17)
Other	0.91 (0.76, 1.09)	0.96 (0.80, 1.16)	0.85 (0.67, 1.06)	0.99 (0.83, 1.18)	1.08 (0.89, 1.29)	1.02 (0.81, 1.28)
Employment						
Employed	Ref.	Ref.	Ref.	Ref.	Ref.	Ref.
Unemployed	1.07 (0.95, 1.21)	1.07 (0.94, 1.21)	1.03 (0.91, 1.18)	2.01 (1.79, 2.25) ***	1.78 (1.57, 2.00) ***	1.74 (1.53, 1.98) ***
IMD						
1 Most deprived	1.07 (0.75, 1.53)	1.16 (0.73, 1.50)	1.10 (0.75, 1.63)	1.43 (0.99, 2.07)	1.12 (0.77, 1.65)	0.90 (0.60, 1.34)
2	0.92 (0.62, 1.36)	0.90 (0.60, 1.34)	0.91 (0.59, 1.39)	1.07 (0.71, 1.62)	1.08 (0.71, 1.63)	1.00 (0.65, 1.55)
3	1.06 (0.73, 1.52)	1.06 (0.73, 1.53)	1.04 (0.70, 1.55)	1.16 (0.79, 1.70)	1.05 (0.71, 1.55)	0.91 (0.61, 1.38)
4	1.23 (0.86, 1.75)	1.24 (0.86, 1.78)	1.18 (0.80, 1.74)	1.35 (0.93, 1.95)	1.17 (0.80, 1.71)	0.96 (0.64, 1.43)
5 Least deprived	Ref.	Ref.	Ref.	Ref.	Ref.	Ref.

^1^Reference group is ‘started treatment’ for both outcomes. * *p* < 0.05; **p < 0.01; *** *p* < 0.001 Model 1 unadjusted, model 2 adjusted for age, diagnosis and baseline symptom severity scores, model 3 adjusted for age, diagnosis, baseline symptom severity scores and all other social status indicators

**Table 3:** Associations between social status indicators and service use outcomes in the treatment phase

	Treatment phase outcome variables^2^					
	Disengaged			Referred elsewhere		
	Model 1	Model 2	Model 3	Model 1	Model 2	Model 3
	RRR (95% CI)	RRR (95% CI)	RRR (95% CI)	RRR (95% CI)	RRR (95% CI)	RRR (95% CI)
Ethnicity						
White	Ref.	Ref.	Ref.	Ref.	Ref.	Ref.
Asian	1.17 (1.04, 1.32) *	1.04 (0.91, 1.18)	0.97 (0.79, 1.18)	1.53 (1.25, 1.86) ***	1.31 (1.07, 1.61) *	1.46 (1.06, 1.99) *
Black	1.30 (1.08, 1.55) **	1.22 (1.02, 1.47) *	1.11 (0.91, 1.35)	0.78 (0.53, 1.14)	0.73 (0.49, 1.07)	0.69 (0.45, 1.04)
Mixed	1.22 (0.93, 1.60)	1.04 (0.79, 1.37)	0.96 (0.72, 1.29)	1.39 (0.89, 2.18)	1.30 (0.82, 2.05)	1.26 (0.77, 2.07)
Other	1.42 (0.97, 2.08)	1.28 (0.87, 1.88)	1.11 (0.72, 1.72)	1.53 (0.81, 2.87)	1.32 (0.70, 2.50)	1.04 (0.47, 2.27)
Sexuality						
Heterosexual	Ref.	Ref.	Ref.	Ref.	Ref.	Ref.
LGB	0.86 (0.62, 1.19)	0.70 (0.50, 0.97) *	0.75 (0.53, 1.06)	1.26 (0.77, 2.08)	1.13 (0.68, 1.88)	1.16 (0.67, 1.99)
Religion						
No religion	Ref.	Ref.	Ref.	Ref.	Ref.	Ref.
Christian	0.90 (0.80, 1.02)	1.03 (0.90, 1.17)	1.01 (0.88, 1.16)	0.90 (0.72, 1.13)	0.92 (0.73, 1.17)	0.95 (0.74, 1.21)
Muslim	1.31 (1.13, 1.52) ***	1.23 (1.06, 1.44) **	1.31 (1.05, 1.62) *	1.22 (0.93, 1.59)	1.04 (0.79, 1.36)	0.77 (0.53, 1.12)
Other	0.76 (0.64, 0.90) **	0.84 (0.70, 0.99) *	0.93 (0.75, 1.15)	1.38 (1.07, 1.79) *	1.37 (1.05, 1.78) *	1.11 (0.79, 1.55)
Employment						
Employed	Ref.	Ref.	Ref.	Ref.	Ref.	Ref.
Unemployed	1.56 (1.40, 1.74) ***	1.36 (1.21, 1.53) ***	1.29 (1.14, 1.46) ***	2.59 (2.16, 3.10) ***	2.09 (1.73, 2.53) ***	2.24 (1.84, 2.75) ***
IMD						
1 (Most deprived)	2.33 (1.65, 3.28) ***	2.17 (1.53, 3.09) ***	1.84 (1.27, 2.66) **	1.43 (0.80, 2.53)	1.32 (0.73, 2.36)	0.97 (0.53, 1.76)
2	1.28 (0.88, 1.87)	1.26 (0.86, 1.85)	1.06 (0.71, 1.59)	1.60 (0.87, 2.94)	1.57 (0.85, 2.90)	1.33 (0.71, 2.50)
3	1.49 (1.05, 2.13) *	1.41 (0.99, 2.03)	1.26 (0.86, 1.83)	1.39 (0.78, 2.49)	1.29 (0.71, 2.32)	0.96 (0.52, 1.75)
4	1.87 (1.32, 2.64) ***	1.72 (1.21, 2.44) **	1.47 (1.02, 2.12)*	1.37 (0.78, 2.44)	1.23 (0.69, 2.20)	0.91 (0.51, 1.65)
5 (Least deprived)	Ref.	Ref.	Ref.	Ref.	Ref.	Ref.

^2^Reference group ‘completed treatment’ for both outcomes. Results based upon analysis of reduced sample (*n* = 6852) because men from the ‘assessment only group’ were removed. *p < 0.05; **p < 0.01; ***p < 0.001. Model 1 unadjusted, model 2 adjusted for age, diagnosis and baseline symptom severity scores, model 3 adjusted for age, diagnosis, baseline severity scores and all other social status indicators

### Assessment phase

Across unadjusted and adjusted models, Asian and Black men were at a higher risk of disengaging following assessment compared to White men (Asian: RRR = 1.32 (95% CI) = 1.07–1.61), *p* = 0.008; Black: RRR = 1.32 (95% CI = 1.07–1.64), *p* = 0.010). LGB men were found to be at increased risk of disengaging than heterosexual men; however, this association was attenuated in the adjusted models (*p* = 0.107). Muslim men were more at risk of disengaging than non-religious men; however, this was also attenuated in the fully adjusted model. Christian men were at a lower risk of disengaging from treatment compared to the non-religious men in all models (RRR = 0.85 (95% CI = 0.72–1.00), *p* = 0.044). In contrast, no associations between socioeconomic indicators and disengagement were identified across models. Black men were at increased risk of being deemed unsuitable compared to White men (RRR = 1.26 (95% CI = 1.01–1.56), as were unemployed men compared to not-unemployed men (RRR = 1.74 (95% CI = 1.53–1.98), *p* < 0.001). No other associations were observed.

### Treatment phase

Having commenced treatment, Black and Asian men were at higher risk of disengaging than completing treatment compared to the White men. However, this was attenuated for both groups in adjusted models. LGB men were at reduced risk of disengagement but only when controlling for age and symptom severity covariates. Muslim men were at increased risk of disengaging compared to non-religious men across all models (RRR = 1.31 (95% CI = 1.05–1.62), *p* = 0.015). Men from Other religious groups were at less risk of disengaging then the non-religious men, but this was attenuated in the fully adjusted model. Unemployed men were at an increased risk of disengaging from treatment (compared to not-unemployed) across all models (RRR = 1.29 (95% CI = 1.14–1.46), *p* < 0.001). Similarly, men living in the most deprived neighbourhoods were at greater risk of disengaging compared to those in the least deprived neighbourhood in all models (RRR = 1.84 (95% CI = 1.27–2.66), *p* = 0.001). Men living in neighbourhoods in second least deprived quintile were also at increased risk of disengaging from treatment (RRR = 1.47 (95% CI = 1.02–2.66), *p* = 0.04). Asian men were found to be at increased risk of being referred elsewhere than completing treatment compared to White men across all models (RRR = 1.46 (95% CI = 1.06–2.00), *p* = 0.019). No association was found between sexual orientation and being referred elsewhere. Men from the other religious groups were at increased risk of being referred elsewhere; however, this association was attenuated in the fully adjusted model. Unemployed men were at increased risk of being referred elsewhere compared to not-unemployed men across all models (RRR = 2.25 (95% CI = 1.84–2.75), *p* < 0.001). No association was found between neighbourhood deprivation level and being referred elsewhere.

### Latent class analysis

Model fit statistics for the LCA and the model selection procedure are presented in Appendix C. A seven-class model was selected as the optimal class solution, with ethnicity and religion appearing to most distinguish between class identities. Descriptive labels were assigned to each class to summarise the prominent characteristics of each in relation to ethnicity and religion. Table 4 presents the seven identified classes.

**Table 4:** Description of latent classes

Class 1	*n* = 3241 (32.7%)	*‘White British, non-religious’* Ethnicity: White British (100%) Religion: non-religious group (100%) Not-unemployed: (67%) Heterosexual: (96%)
Class 2	*n* = 665 (6.7%)	‘ *Minoritised ethnic, non-religious’* Ethnicity: Asian (38%), Black (32%), Mixed (22%), Other (8%) Religion: non-religious group (100%) Not-unemployed: (61%) Heterosexual: (95%)
Class 3	*n* = 1782 (18%)	*‘Asian, Muslim’* Ethnicity: Asian (80%), White (8%), Black (5%), Mixed (3%) Other (4%) Religion: Muslim (100%) Not-unemployed: (62%) Heterosexual: (97%)
Class 4	*n* = 2082 (21%)	*‘White British, Christian’* Ethnicity: White British (100%) Religion: Christian (100%) Not-unemployed: (69%) Heterosexual: (98%)
Class 5	*n* = 820 (8.3%)	‘*Asian, other religion’* Ethnicity: Asian (100%) Religion: other religion (100%) Not-unemployed: (71%) Heterosexual: (99%)
Class 6	*n* = 474 (4.8%)	*‘White British, other religion’* Ethnicity: White British (80%), Black (7%), Mixed (6%) Other (6%) Religion: other religion (100%) Not-unemployed: (74%) Heterosexual: (96%)
Class 7	*n* = 840 (8.5%)	*‘ Minoritised ethnic, Christian’* Ethnicity: Black (67%), Asian (12%), Mixed (18%), Other (3%) Religion: Christian (100%) Not-unemployed: (65%) Heterosexual: (98%)

### Association between class and service use

The association between the classes identified in the LCA were then tested for association with the service use outcomes using multinomial regression analysis. The results are presented in Table 5 and described below.

**Table 5:** Associations between class and service use outcomes

		Assessment phase^a^			
		Disengaged		Service deemed unsuitable	
		Model 1	Model 2	Model 1	Model 2
	*n*	RRR (95% CI)	RRR (95% CI)	RRR (95% CI)	RRR (95% CI)
Class 1	(3241)	Ref.		Ref.	Ref.
Class 2	(665)	1.44 (1.15, 179)**	1.29 (1.02, 1.63)*	1.19 (0.94, 1.50)	1.21 (0.95, 1.54)
Class 3	(1782)	1.45 (1.24, 1.69)***	1.41 (1.20,1.66)***	1.11 (0.94, 1.30)	1.17 (0.98, 1.39)
Class 4	(2082)	0.77 (0.65, 0.91)**	0.89 (0.75, 1.07)	0.98 (0.84, 1.15)	0.98 (0.83, 1.16)
Class 5	(820)	1.13 (0.91, 1.40)	1.16 (0.93, 1.45)	1.15 (0.93, 1.42)	1.24 (0.99, 1.55)
Class 6	(474)	0.84 (0.63, 1.33)	1.02 (0.76, 1.38)	0.95 (0.72, 1.24)	1.11 (0.82, 1.48)
Class 7	(840)	1.14 (0.92, 1.42)	1.15 (0.92, 1.43)	1.18 (0.96, 1.45)	1.21 (0.97, 1.51)

		Treatment phase^b^			
		Disengaged		Referred elsewhere	
		Model 1	Model 2	Model 1	Model 2
	*n*	RRR (95% CI)	RRR (95% CI)	RRR (95% CI)	RRR (95% CI)
Class 1	(3241)	Ref.	Ref.	Ref.	Ref.
Class 2	(665)	1.23 (0.99, 1.53)	1.15 (0.92, 1.44)	1.27 (0.87, 1.86)	1.11 (0.74, 1.64)
Class 3	(1782)	1.38 (1.19, 1.60)***	1.31 (1.12, 1.53)**	1.28 (0.98, 1.67)	1.09 (0.82, 1.44)
Class 4	(2082)	0.93 (0.80, 1.06)	1.06 (0.91, 1.23)	0.94 (0.73, 1.22)	0.91 (0.70, 1.20)
Class 5	(820)	0.78 (0.63, 0.97)*	0.86 (0.69, 1.07)	1.73 (1.28, 2.34)***	1.60 (1.17, 2.19)**
Class 6	(474)	0.90 (0.70, 1.15)	1.17 (0.90, 1.53)	1.14 (0.75, 1.73)	1.21 (0.78, 1.87)
Class 7	(840)	1.12 (0.92, 1.37)	1.12 (0.92, 1.38)	1.07 (0.75, 1.53)	1.00 (0.69, 1.45)

*p < 0.05; **p < 0.01; ***p < 0.001. Model 1 unadjusted, model 2 adjusted for age, diagnosis, baseline severity scores and neighbourhood deprivation level.

^a^Reference group started the treatment

^b^Reference group completed the treatment

Following assessment, men in the minoritised ethnic, non-religious group (class 2) and Asian, Muslim group (class 3) were at increased risk of disengaging from treatment rather than starting treatment compared to those in the White British, non-religious group (class 1) (Class 2: RRR = 1.29 (95% CI = 1.02;1.63), *p* = 0.031; Class 3: RRR = 1.41 (95% CI = 1.20;1.66), *p* < 0.001). In contrast, White British Christian men (class 4) were at lower risk of disengagement than starting treatment, compared to those in the White non-religious group; however, this was attenuated in the adjusted model. No associations were found between class groups and the service deemed unsuitable group.

Having commenced treatment, men in the Asian, Muslim group (class 3) were at increased risk of disengaging from treatment rather than completing treatment, compared to men in the White British, non-religious group (class 1) (RRR = 1.31 (95% CI = 1.12;1.53), *p* = 0.001). Men from the Asian, other religion group (class 5) were found to be at lower risk of disengaging than completing treatment, but this was attenuated in the adjusted model. Men from the Asian, other religion group (class 5) were at increased risk of being referred elsewhere rather than completing treatment compared to men from the White British, non-religious group (class 1) (RRR = 1.60 (95% CI = 1.17–2.19), *p* = 0.003).

## Discussion

Drawing from a large diverse sample, this study compared approaches to understanding the intersectional impact of sociodemographic and socioeconomic factors associated with men’s mental health service use using two different statistical methods. Findings from the initial regression analyses reveal considerable disparities in relation to the mental health service use of men. Being from a minoritised ethnic group, being Muslim, being unemployed, and living in a deprived neighbourhood were associated with a discontinuation of service contact. These results are consistent with previous research findings which suggest that being from a minoritised ethnic group and being of lower socioeconomic status are associated with lower levels of service use [13–17]. However, the results also go further to reveal that religious group, a characteristic rarely explored, is an important sociodemographic characteristic for men’s mental health service use. That men self-identifying as Christian are less likely to disengage following assessment and those self-identifying as Muslim are more likely to disengage after commencing treatment suggests that disaggregating by religious group, rather than just religiosity, may be important where possible.

Seven subgroups of men were identified in the LCA, providing a more detailed picture of the intersectional impact of social determinants on service use. For example, where there was an association between identifying as Black or Asian and a higher risk of disengagement following assessment in the regression models, in the LCA analysis it was apparent that both Asian Muslim men and men who are both non-religious and from a minoritised ethnic group were more likely to disengage following assessment. However, little differences between classes with regard to employment status, which was an important predictor of outcomes in the initial regression models, might suggest that a potentially important source of disadvantage could be overlooked if the LCA had been the sole analytic model used.

### Limitations

Data were drawn from two services in North East London, therefore inclusion of additional services would be needed for generalisability. It is possible that patterns of disparities will differ according to the composition of the local populations served and by organisational features of individual services, including the priority given to adapting practice to promote inclusivity. This study was limited to using variables derived from routinely collected data; there are several other unmeasured social demographic characteristics, such as migrant status and relationship status, that might be of value to explore in future work. Although one of the strengths of this study stems from the use of an ethnically diverse dataset, the need to ensure sufficient power to detect effects and to optimise the chances of model convergence meant that ethnicity categories with known heterogeneity remained, by necessity, broad. Similarly, additional measures of SES (e.g. housing status, educational attainment, and income levels) could have better reflected the complexity of the construct [54]. It is acknowledged that the unemployed and not-unemployed groups used here encompassed a considerable range of circumstances. The outcome data were based upon the reasons clinicians assigned for end of treatment episode; future research is needed which explores this topic from the service user perspective. Finally, 6% of the total sample did not have outcome data and were therefore excluded. Differences were observed between the excluded group and those included, such as a higher likelihood of being Asian, being Muslim and residing in more deprived neighbourhoods in the group with missing outcome data. Exploring the cause of this was beyond the scope of the current analysis but it may be of interest to services to investigate, potentially at the case-note level, why these data were not available. Using LCA it was not possible to provide a full insight into the intersectional relationships between the full range of social status variables used, with the class solutions offering little differentiation by sexual orientation or employment status. This may reflect higher levels of uncertainty in the data than anticipated or a challenge inherent to using power-based statistics to detect intersectional groups which, by their nature, will have smaller case numbers than the larger categories they are derived from. Given that only 3% of the sample identified as non-heterosexual, it is perhaps unsurprising that this group did not feature in the classes. Whilst the 7-class solution was the best fit using the available metrics and provided a more clinically relevant grouping of service users, it may be that LCA was not the optimum approach using these variables, especially given the low frequency of some categories. Whilst LCA has been used on similar samples and variables [29, 47], future research might explore alternative classification procedures such as CART or machine learning classification algorithms which might offer new ways of identifying sub-groups based on lower frequency differences, but this is yet to be demonstrated in the field.

### Research and clinical implications

The results demonstrate disparities which vary by outcome, highlighting the importance of refining service use measures where possible. Failure to differentiate between clinician or service user led decision-making and stage along the treatment pathway may have led to certain subgroups at risk of discontinuing treatment remaining hidden. Furthermore, the action needed to reduce disengagement will be different from that required to better understand and rectify possible disparities in clinician-led decisions to discontinue treatment.

‘Clinician-led decisions’, reflect not only the judgement and experience of an individual therapist but the values and possibilities afforded by the organisation in which they are embedded. As such, action to reduce disparities is required at all levels. Rates of disengagement can be reduced for underserved groups such as men and minoritised ethnicity groups by adapting services to meet their specific needs [35–37, 55]. Recent clinical guidelines have been developed by IAPT services aiming to improve access and outcome equity for minority ethnic IAPT service users [56]. Suggestions to improve engagement levels of minoritized ethnic groups include increasing workforce diversity, ensuring adequate staff training and supervision in cross-cultural competence and offering culturally adapted and culturally responsive therapies [56]. Culturally adapted care has also been shown to improve outcomes for minoritised ethnicity groups, particularly where adaptations also occur at the organisational level, for example, improved access via community outreach or providing locations deemed more appropriate by target groups [57]. The results of the current study justify the expansion of current clinical guidelines focussed upon minoritised ethnicity groups to include consideration of the needs of men, particularly Muslim and socio-economically disadvantaged men. However, the intersectional nature of the results complicates the nature of this task; caution is needed to ensure that in organising clinical guidance according to different facets of social identity there is not an inadvertent, homogenising of other important social characteristics that may exist within the group. Greater risk of disengagement for some subgroups may stem from the lived experience of occupying multiple stigmatised identities, which may be inadequately captured if facets of social identity are treated independently. It is important therefore that the current momentum towards developing guidance for culturally adapted services is balanced against an equal emphasis of the continued need to assess and work flexibly with each client’s uniquely differing values and needs [58].

The disparities found in disengagement levels may also be reduced were clinicians to invest extra time discussing, with the most ‘at-risk’ service users, the benefits of staying the course of treatment. Normalising the challenges that can be encountered when attending therapy and providing space for service users to voice any concerns they have could enable joint problem solving between clinician and service user to tackle perceived barriers to engagement.

It was found that certain groups of men were more likely to be perceived by clinicians as having needs better met elsewhere. This constitutes a less explored source of disparity which, based upon the results here, warrants further attention. Unfortunately, whilst service suitability is routinely discussed and clinical reasoning is detailed in individual records, it is not coded in a format conducive to inclusion in large scale quantitative analysis. In future, services may want to consider including a coded measure to better understand such disparities. There are several possible explanations. It may be that, despite controlling for diagnosis and symptom severity levels, residual confounding due to unmeasured aspects of the clinical presentations (e.g. chronicity of illness or substance use comorbidity) could explain the effect or some proportion of it. It is plausible that being unemployed may in this context be a proxy marker of increased complexity of need. It may also be that diagnostic labels, which exclude service users from IAPT treatment, such as bipolar disorder or depression with psychotic symptoms, are being applied to men from minoritised ethnicity backgrounds at greater rates, reflective of a broader trend described in the literature of higher incidence of psychosis and bipolar disorder found in minoritised ethnicity groups [59, 60]. However, given that service users who, following the initial assessment, were referred to another clinical service for treatment were *not* included in the ‘service deemed unsuitable’ group, these hypotheses only apply to the ‘referred elsewhere’ group. More relevant to the ‘service deemed unsuitable’ group may be some form of associated systematic bias leading to discontinuation for this reason, such as requiring sessions in the evenings or at weekends, which may not have been possible. Finally, it may be that certain groups of service users may be perceived as more ‘difficult to engage’, perhaps due to cultural variations in the expression of distress less familiar to the clinician or due to inhibited ability to express distress due the intersection of socialised notions of masculinity with other aspects of their identity. More research is needed which investigates the reasons behind the disparities in the outcomes found here, ideally by focussing on service users who declined treatment altogether.

## Supplementary Information

Below is the link to the electronic supplementary material.

Supplementary file1 (DOCX 47 KB)

## Data Availability

This dataset contains personal National Health Service information and is not made routinely available.

## Code availability

Mplus code is available upon request to the corresponding author.
