# Supplementary material for: Understanding differences in mental health service use by men: an intersectional analysis of routine data
Source: Soc Psychiatry Psychiatr Epidemiol. 2022 Mar 22;57(10):2065–77. doi: 10.1007/s00127-022-02256-4 (PMC9477949; doi:10.1007/s00127-022-02256-4)
Supplement: Supplementary file 1 — Supplementary file1 (DOCX 47 KB) [file 127_2022_2256_MOESM1_ESM.docx]

Supplementary materials to:

**Understanding differences in mental health service use by men: an intersectional analysis of routine data**

Content:

Appendix A: Comparison of service users with and without outcome data. (Page 2)

Appendix B: Participant flow diagram. (Page 3)

Appendix C: Latent Class Analysis. (Page 4)

**Appendix A: Comparison of service users with and without outcome data**

**Table S1: Comparison of samples.**

|  | Study sample | Missing DV data |  |
| --- | --- | --- | --- |
|  | *n* (%) | *n* (%) | *p* |
| Total sample | 9, 904 (94%) | 637 (6%) |  |
|  |  |  |  |
| **Age** (years) |  |  |  |
| 16-24 | 1,513 (15.3%) | 112 (17.6%) | 0.025 |
| 25-34 | 2,665 (26.9%) | 177 (27.7%) |  |
| 35-44 | 2,206 (22.3%) | 162 (25.4%) |  |
| 45-54 | 1,903 (19.2%) | 100 (15.7%) |  |
| 55-64 | 1,109 (11.2%) | 55 (8.6%) |  |
| 65+ | 507 (5.1%) | 32 (5.0%) |  |
|  |  |  |  |
| **Ethnicity** |  |  |  |
| White | 5,769 (58.3%) | 280 (43.9%) | <0.001 |
| Asian | 2,597 (26.2%) | 253 (39.7%) |  |
| Black | 902 (9.1%) | 49 (7.7%) |  |
| Mixed | 370 (3.7%) | 25 (3.9%) |  |
| Other | 184 (1.9%) | 21 (3.3%) |  |
| Missing | 81 (0.8%) | 10 (1.6%) |  |
|  |  |  |  |
| **Sexuality** |  |  |  |
| Heterosexual | 9,234 (93.2%) | 573 (89.8%) | 0.001 |
| Non-heterosexual | 291 (2.9%) | 22 (3.5%) |  |
| Missing | 378 (3.8%) | 43 (6.7%) |  |
|  |  |  |  |
| **Religion** |  |  |  |
| No religion | 3,609 (36.4%) | 238 (37.3%) | <0.001 |
| Christian | 2,910 (29.4%) | 128 (20.1%) |  |
| Muslim | 1,695 (17.1%) | 154 (24.1%) |  |
| Other | 1,294 (13.1%) | 101 (15.8%) |  |
| Missing | 395 (4.0%) | 17 (2.7%) |  |
|  |  |  |  |
| **Employment** |  |  |  |
| Employed | 6, 502 (65.7%) | 436 (68.3%) | 0.056 |
| Unemployed | 3,288 (33.2%) | 189 (29.6%) |  |
| Missing | 113 (1.1%) | 13 (2.0%) |  |
|  |  |  |  |
| **IMD** (quintiles) |  |  |  |
| 1 (Least deprived) | 271 (2.7%) | 25 (3.9%) | <0.001 |
| 2 | 870 (8.8%) | 88 (13.8%) |  |
| 3 | 1,915 (19.3%) | 167 (26.2%) |  |
| 4 | 3,301 (33.3%) | 209 (32.8%) |  |
| 5 (Most deprived) | 3,277 (33.1%) | 124 (19.4%) |  |
| Missing | 269 (2.7%) | 25 (3.9%) |  |
|  |  |  |  |
| **Baseline severity score** |  |  |  |
| PHQ-9 mean (SD) | 15.6 (6.4) | 14.8 (6.5) | 0.002 |
| Missing | 51 (0.5%) | 9 (1.4%) |  |
|  |  |  |  |
| GAD-7 mean (SD) | 13.7 (5.2) | 13.1 (5.5) | 0.011 |
| Missing | 59 (0.6%) | 9 (1.4%) |  |
|  |  |  |  |

*p* values calculated using Pearson’s χ^2^ test for social statis indicators. *p* values for symptoms scores were calculated using independent group t tests.

**Appendix B: Participant flow diagram**

| Referred to the services  n = 55, 214 |
| --- |

| Exclusions  n = 34,295: Female service users  n = 2,835: Receiving treatment from March 2020 onwards  n = 5,403: Never attended an assessment  n = 2,135: Not the first episode of care  n = 637: No outcome data available  n = 5: ‘Service user deceased’ as reason for ending use of service |
| --- |

| n = 6,852: Started treatment |
| --- |

| Included in assessment phase analysis  n = 9,904 |
| --- |

| n = 1,483: Disengaged following initial assessment |
| --- |

| n = 1,568: Service deemed unsuitable |
| --- |

| Included in treatment phase  analysis  n = 6,852 |
| --- |

| n = 3,899: Completed treatment |
| --- |

| n = 2,394: Disengaged having commenced treatment |
| --- |

n = 559: Referred elsewhere

**Figure S1: Participant flow diagram.**

**Appendix C: Latent Class Analysis**

Latent class analysis (LCA) is a person-centred clustering (mixture modelling) approach to identify homogeneous sub-groups within a sample [1]. Decisions about the optimal class solution are made using a range of goodness of fit statistics, using criteria established by exports and used in previous research [2]–[4]. The model fit statistics considered where the Akaike information criterion (AIC), Bayesian information criterion (BIC) sample-size-adjusted Bayesian information criteria (SABIC), entropy and the Vuong-Lo-Mendell-Rubin likelihood ratio test (VLMR-LRT). Comparatively lower AIC, BIC and SABIC values indicate better fit in LCA models. The entropy value ranges between 0 and 1 and measures classification accuracy, with values closer to 1 indicating better classification. The VLMR-LRT statistic compares model solutions. A p-value of > 0.05 indicates that the previous class solution fits the model better than the current one. To ensure results were clinically meaningful and numerically stable, the conventional restriction of needing at least 5% of service users in a class was also used in decision making [2], [4], [5]. As there was no prior hypothesis regarding the number of expected classes, the analytical plan was to begin by conducting the LCA with a two-class solution, assessing it against the fit statistics and then increasing the number of classes until one or more of the parameters for best fit had been met.

Goodness of fit statistics for the LCA are presented in Table S2. The AIC, BIC and SABIC values continued to diminish and the VLRT p-value remained significant (p<0.05) from the 2- to 10-class solutions. Entropy was high (>0.968) for all solutions. However, the 8-class solution (as well as 9- and 10-class) identified a class that included <4% of the sample, and therefore the 7-class solution was selected.

**Table S2: Goodness of fit statistics for LCA models**

| Class solution | Model fit statistics | | | | | |
| --- | --- | --- | --- | --- | --- | --- |
|  | AIC | BIC | SABIC | E | LMR-LRT  p-value | % per class |
|  |  |  |  |  |  |  |
| 2 class | 78241.158 | 78406.774 | 78333.683 | 0.993 | <0.001 | 41/59 |
| 3 class | 70927.880 | 71179.905 | 71068.680 | 0.968 | <0.001 | 37/34/30 |
| 4 class | 66579.103 | 66917.536 | 66769.177 | 0.974 | <0.001 | 30/13/18/39 |
| 5 class | 63903.408 | 64328.249 | 64140.756 | 0.978 | <0.001 | 21/13/18/33/15 |
| 6 class | 61575.343 | 62086.592 | 61860.964 | 0.978 | <0.001 | 8/21/18/7/33/13 |
| **7 class** | **60265.687** | **60863.344** | **60599.583** | **0.979** | **<0.001** | **8/7/18/21/8/5/33** |
| 8 class | 59048.848 | 59732.914 | 59431.018 | 0.979 | <0.001 | 5/33/7/8/8/15/21/4 |
| 9 class | 58345.413 | 59115.887 | 58775.857 | 0.980 | <0.001 | 6/5/21/3/15/33/6/8/4 |
| 10-Class | 57885.019 | 58741.902 | 58363.738 | 0.981 | 0.003 | 8/5/6/4/21/15/33/4/3/3 |

**References**

[1] R. Saunders, J. E. J. Buckman, and S. Pilling, “Latent variable mixture modelling and individual treatment prediction,” *Behav. Res. Ther.*, vol. 124, no. June 2019, p. 103505, 2020, doi: 10.1016/j.brat.2019.103505.

[2] P. Spinhoven, N. Batelaan, D. Rhebergen, A. van Balkom, R. Schoevers, and B. W. Penninx, “Prediction of 6-yr symptom course trajectories of anxiety disorders by diagnostic, clinical and psychological variables,” *J. Anxiety Disord.*, vol. 44, pp. 92–101, 2016, doi: 10.1016/j.janxdis.2016.10.011.

[3] K. L. Nylund, T. Asparouhov, and B. O. Muthén, “Deciding on the number of classes in latent class analysis and growth mixture modeling: A Monte Carlo simulation study,” *Struct. Equ. Model.*, vol. 14, no. 4, pp. 535–569, 2007, doi: 10.1080/10705510701575396.

[4] R. Saunders, J. Cape, P. Fearon, and S. Pilling, “Predicting treatment outcome in psychological treatment services by identifying latent profiles of patients,” *J. Affect. Disord.*, vol. 197, pp. 107–115, 2016, doi: 10.1016/j.jad.2016.03.011.

[5] Y. Yuan, H. S. Min, K. L. Lapane, A. J. Rothschild, and C. M. Ulbricht, “Depression symptoms and cognitive impairment in older nursing home residents in the USA: A latent class analysis,” *Int. J. Geriatr. Psychiatry*, vol. 35, no. 7, pp. 769–778, 2020, doi: 10.1002/gps.5301.
